# Guidewire Mishap: An Avoidable Iatrogenic Complication

**DOI:** 10.5005/jp-journals-10071-23225

**Published:** 2019-08

**Authors:** Archana Rani, Praveen K Malik

**Affiliations:** 1 Department of Anesthesia, Hamdard Institute of Medical Sciences and Research, New Delhi, India; 2 Department of Medicine, ESIC Medical College and Hospital, Faridabad, Haryana, India

**Keywords:** Central venous catheterization, Guidewire, Hemodialysis

## Abstract

Central vein catheterization is a commonly used technique in indoor patients. It is mainly used for venous access, allowing administration of drugs, fluids, chemotherapy, blood components. Central vein catheterisation may lead to many complication which includes bleeding, perforation of veins and hematoma formation, thrombosis, infections, bleeding, needle stick injury, air embolism, kinking or looping of the wire tip, breakage of a guidewire, etc. We, hereby, report a case where the guidewire was lost during femoral venous catherisation. A whole guidewire embolization is completely preventable complication. It may not necessarily produce any symptoms and may remain unnoticed for long time.

**How to cite this article:**

Rani A, Malik PK. Guidewire Mishap: An Avoidable Iatrogenic Complication. Indian J Crit Care Med 2019;23(8):382–383.

## INTRODUCTION

Modified Seldinger technique has been used extensively for insertion of intravascular cannula safely. Being an invasive technique, it is not free of complications. Migration of guidewire either as a whole or fractured tip is rare. The former is a completely preventable complication. Majority of the reported cases of such mishaps are related to central line placement. Although hemodialysis (HD) is considered to be a safe procedure in the hands of experienced persons, we present here a case wherein a guide- wire got misplaced during femoral catheterisation.

## CASE DESCRIPTION

**Fig. 1:**
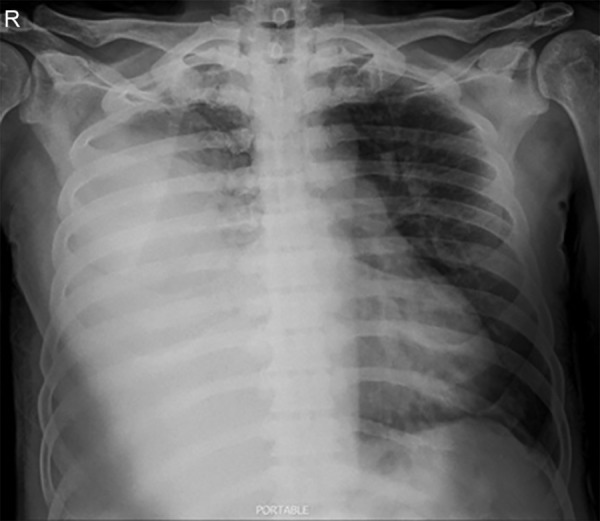
Chest X-ray PA view showing the massive right sided pleural effusion

**Fig. 2:**
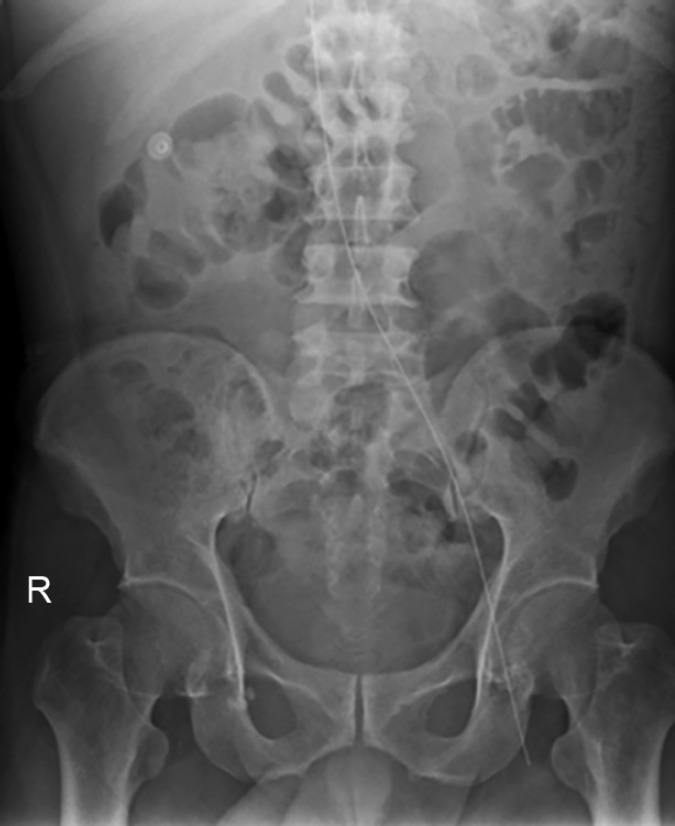
X-ray abdomen and pelvis showing the migrated guidewire in situ with distal end lying in femoral vein

**Fig. 3:**
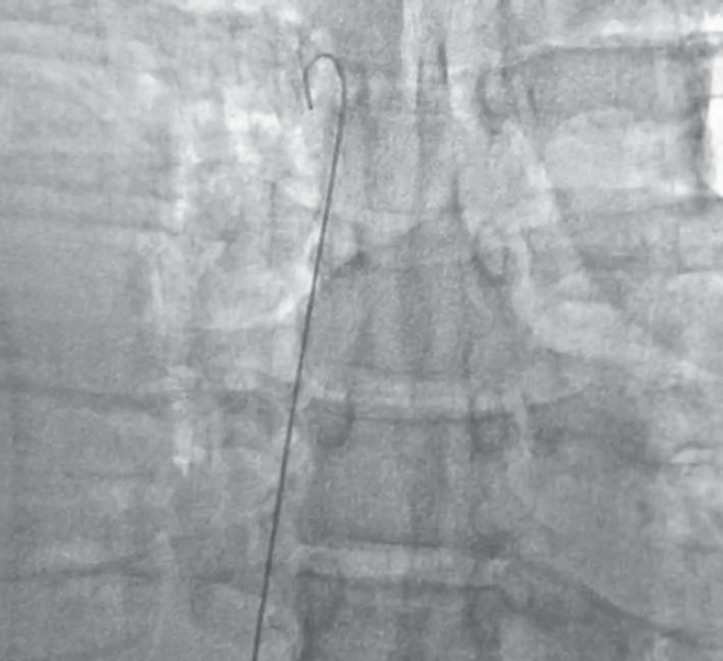
Fluoroscopic image exploring the upper J-end of guidewire, which is lying at the level of T5, corresponding to tight atrium

**Fig. 4:**
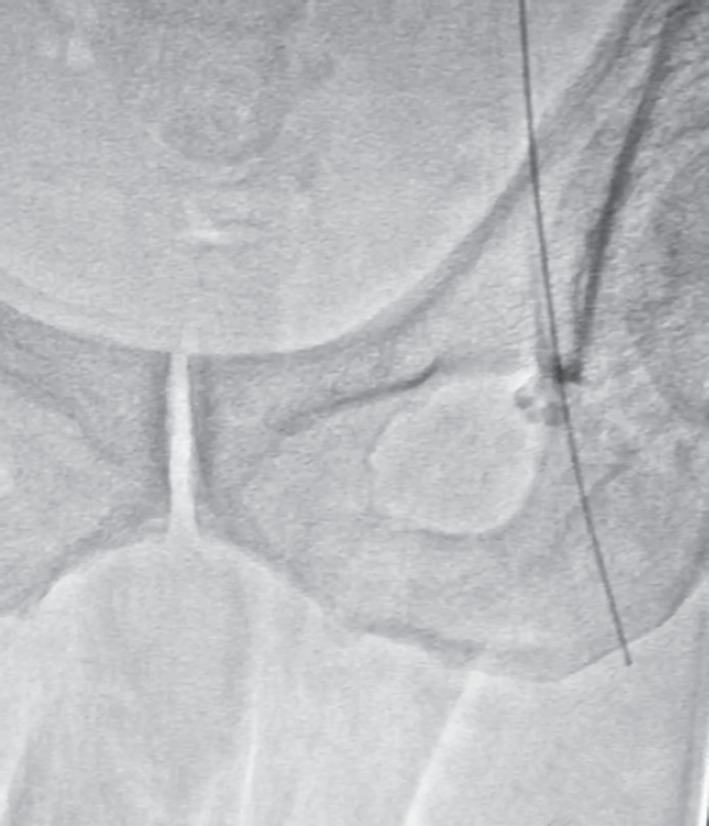
Fluoroscopic image confirming the superficial position of distal end of guidewire in femoral vein just before surgical retrieval

A 65 years old male, who had been suffering from type 2 diabetes mellitus for 13 years and recently diagnosed as a case of end stage renal disease (ESRD), was admitted with dyspnea in rest, pain right side of chest and swelling over whole body. He had stable vitals and pallor, pitting pedal edema, and features of right sided pleural effusion were present. Baseline lab parameters showed severe anemia (Hb 7.5 g%), high kidney function test (blood urea 185 mg/dL, serum creatinine 11.5 mg/dL and K 5.2 mmol/L). X-ray chest showed right sided massive pleural effusion (Fig. 1). Patient was advised HD. Dialysis technician of about 15 years experience performed the femoral catheterization alone. While dilator was pushed, guidewire was also pushed too far inside. Distal end of guidewire could not be retrieved after dilator was withdrawn and created a panic in dialysis room. X-ray abdomen and pelvis was obtained immediately and noticed the migrated guidewire in situ (Fig. 2). Fluoroscopy showed the proximal J-end to be lying at the level of T5-T6 (Fig. 3), and distal end lying very superficially in left femoral vein (Fig. 4). It was retrieved surgically.

## DISCUSSION

Modified Seldinger technique has overall complication rate of 12%.^1^ Various complications reported include perforation of veins and hematoma formation, thrombosis, infections, bleeding, needle stick injury, air embolism, kinking or looping of the wire tip, breakage of a guide wire etc. A whole guidewire embolization is completely preventable complication. It may not necessarily produce any symptoms and may remain unnoticed for long time.^2^ However, intravascular migration of a broken guidewire has the potential of causing adverse effects with fatality rate of up to 20%.^3^ Complications of prolonged retention of guidewire in situ include thrombosis, infections (septic thrombophlebitis, endocarditis), pulmonary embolism, arrhythmias, cardiac and vascular damages.^4^ Once diagnosed, it should be removed immediately to prevent such complications. Percutaneous approach using vascular snares under fluoroscopic guidance should be the first choice.^5^ In our case, the distal tip lied very superficial and could be easily removed surgically. Surgical removal is also indicated when the percutaneous approach fails or where such facilities are not available.^6^ Certain precautions and tips are noteworthy to avoid such completely preventable complications. Catheter and guide- wire should never be advanced together into the vein. One should make sure that the wire is visible at the outer end before the dilator or catheter is advanced.^2^ The wire should always be inspected to ensure complete removal at the end of the procedure.^7^ An artery forceps may be used to clamp the outer end of wire in order to prevent the inadvertent migration.^8^
